# Treatment of oral dysfunction

**DOI:** 10.1186/1746-160X-8-S1-I14

**Published:** 2012-05-25

**Authors:** Anita McAllister

**Affiliations:** 1 Department of Clinical and Experimental Medicine, Unit for Speech and Language Pathology, Linköping University, Sweden

## 

The orofacial area is central for several vital functions such as breathing and eating. The orofacial area also acts as the basis for social interaction, emotional communication, facial expression and speech communication. Normal orofacial sensorimotor development is necessary for these functions to develop and mature in the child, hence orofacial dysfunction may be severely disabling.

In the oral cavity, saliva lubricates the hard and soft tissues. Lack of saliva impairs speech clarity, chewing and swallowing, taste and voice quality. The complexity of these functions means that several clinical professionals, such as dentists, gastroenterologists, ENT specialists, phoniatricians or laryngologists, and speech and language pathologists, are needed to address the problems. Through a multiprofessional Nordic cooperation an assessment instrument for orofacial functions was developed, the Nordic Orofacial Test-Screening (NOT-S). In a previous study, 46 individuals with ectodermal dysplasia (ED) were investigated using NOT-S. The most frequently recorded dysfunctions were in the domains chewing and swallowing (82.6%), dryness of the mouth (45.7%) and speech (43.5%). Also hoarseness, a parameter that was added to the test, was found in 32.6% of individuals with ED. The treatment alternatives for these problems include above mentioned specialists and a number of different treatment options, such as surgery to improve anatomical conditions, artificial saliva to lubricate the oral mucosa, sensorimotor training to improve chewing and swallowing, or speech and language therapy to address problems with speech production or voice quality.

